# Limb Ischemic Preconditioning Protects Endothelium from Oxidative Stress by Enhancing Nrf2 Translocation and Upregulating Expression of Antioxidases

**DOI:** 10.1371/journal.pone.0128455

**Published:** 2015-06-01

**Authors:** Min Chen, Mingsheng Zhang, Xuanping Zhang, Jie Li, Yan Wang, Yanying Fan, Ruizan Shi

**Affiliations:** Department of Pharmacology, Shanxi Medical University, Xinjiannanlu 56, Taiyuan, 030001, Shanxi Province, China; Massachusetts General Hospital/Harvard Medical School, UNITED STATES

## Abstract

Remote ischemic preconditioning is often performed by limb ischemic preconditioning (LIPC), which has been demonstrated to be beneficial to various cells, including endothelial cells. The mechanisms underlying the protection have not been well clarified. The present study was designed to observe the effects of sera derived from rats after LIPC on human umbilical vein endothelial cells (HUVECs) injured by hydrogen peroxide (H_2_O_2_) -induced oxidative stress and explore the involvement of redox state in the protection. Incubation with 1 mM H_2_O_2_ for 2 h induced a significant reduction in HUVECs’ viability with increased production of malondialdehyde (MDA) and reactive oxygen species (ROS). Preincubation with early preconditioning serum (EPS) or delayed preconditioning serum (DPS) derived from rats subjected to LIPC alleviated these changes. Both EPS and DPS increased the nuclear translocation of transcription factor nuclear factor E2-related factor 2 (Nrf2) and the expression of antioxidases. The protective effects of EPS and DPS were blocked neither by MEK/ERK inhibitors U0126 nor by PI3K/Akt inhibitors LY294002. In conclusion, the present study provides the evidence that LIPC protects the HUVECs from H_2_O_2_-induced injury by, at least partially, enhancement of Nrf2 translocation and upregulation of antioxidases via signaling pathways independent of MEK/ERK and PI3K/Akt.

## Introduction

Remote ischemic preconditioning is effective in mitigating injuries induced by both ischemia-reperfusion (I/R) and other hazardous factors in remote tissues or organs such as heart, liver, kidney, brain and intestine [1–6]. It is in most cases performed by effective, feasible, cost-effective and adverse-effect-free limb ischemic preconditioning (LIPC). The substances and the mechanisms underlying the protection have not been well clarified, although a variety of mechanisms [7–9].

Perfusion with the effluent collected from a preconditioned isolated rabbit heart alleviated I/R injury in the non-preconditioned isolated heart and transfusion of blood from a preconditioned rabbit into a non-preconditioned rabbit reduced I/R injury [10]. Serum derived from patients after LIPC reduced hypoxia-induced cell damage in cultured human intestinal cells via inhibition of matrixmetalloproteinase -2 and -9 [4]. Upregulation of heme oxygenase-1 expression and antioxidative effect was suggested responsible for LIPC-induced protection on injured organs including heart [5, 11] and liver [6]. These studies suggest that the protective substances are produced upon LIPC, released to serum and transported through blood to convey the protection on the remote jeopardized parenchymal cells. It was suggested that the humoral factors (hydrophobic and less than 15 kDa) were responsible for the protection which is transferable across species [12].

Repair, rejuvenation and regeneration of injured parenchymal cells depend on the local blood supply. Blood vessels and vascular cells play an irreplaceable role in mediating and/or translating effects of released bioactive substances on the remote injured cells. Endothelial cells are especially important, because they serve as a paracrine system in regulating other cells both in vasculature and in the parenchymal cells via cross-talking mechanisms. Moreover, the endothelium itself may be both a vital target and amplifier for biologic responses to circumstance changes including I/R. Therefore, it can be supposed that the responses of vascular cells to LIPC may, at least partially, mediate LIPC-induced protection.

Effectiveness of LIPC in improving endothelial functions has been proved in human volunteer of I/R subjects [1–3], healthy and hypertensive subjects [13], intensive-exercise subjects [14] and subjects receiving percutaneous coronary intervention [15]. These studies mainly demonstrated that LIPC improves endothelium-dependent vasodilation, but little attention has been paid to how LIPC affects the vascular endothelial cells biochemically and biophysically. A better understanding of the underlying mechanisms is a prerequisite for proper clinic uses of LIPC. The present study was designed to investigate whether LIPC can prevent endothelial cells from oxidative stress injury, and if so, what are the mechanisms underlying the protection.

## Materials and Methods

### Animals

Male Sprague-Dawley rats (250–300 g, 8 weeks old, provided by Animal Facility Center of Shanxi Medical University, China) were used. The protocols and procedures described in the present experiments were approved by the Animal Care and Use Committee of Shanxi Medical University and conformed to the Guide for the Care and Use of Laboratory Animals published by the US National Institutes of Health (NIH Publication, 8th Edition, 2011).

### Animal groups, LIPC induction and serum collection

Twenty four male Sprague-Dawley rats were randomly divided into sham preconditioning, early preconditioning and delayed preconditioning groups. The rats were housed in constant condition (temperature 22 ± 2°C and humidity 50%-60%) in a 12 h-light/12 h-dark cycle for 2 weeks as an acclimatization period before the experiments. **A** modified noninvasive blood pressure radiometer cuff was placed around the right hind-limb of the rats. LIPC was performed by three cycles of 5 min ischemia (inflated the cuff around the arteria femoralis to 200 mmHg) followed by 5 min reperfusion (deflated the cuff). Sera were derived from rats with sham LIPC (non-preconditioning rat serum, NPS), 20 min after LIPC (early preconditioning serum, EPS) and 24 h after LIPC (delayed preconditioning serum, DPS). Animals were anesthetized with intraperitoneal administration of sodium pentobarbital (40 mg/kg) before the blood was collected from abdominal aorta. Sera were obtained by centrifuging the blood at 4°C, aliquoted to 50 μl each tube and kept at -80°C before addition to the culture medium. Blood gases and electrolytes were measured to make sure that the rats were in good conditions without either acidosis or hyperkalaemia.

### Cell culture

The human umbilical vein endothelial cells (HUVECs) were purchased from American Type Culture Collection (Manassas, VA, USA). HUVECs were cultured in medium composed of Dulbecco’s modified Eagle’s medium (Invitrogen Life Technologies, Carlsbad, CA, USA) containing 4.5 g/L D-glucose, 2.5 mM L-glutamine, 110 mg/L sodium pyruvate, 100 U/ml penicillin/streptomycin, 0.125 mg/L amphotericin B, and 10% heat-inactivated fetal calf serum (Invitrogen Life Technologies, Carlsbad, CA, USA) in a humidified incubator with 5% CO_2_ at 37°C. HUVECs were not used in the experiments until they had reached 80% confluence.

The HUVECs were divided into 5 groups. Control: cultured with normal medium without any intervention throughout the experiment. Model: cultured with normal medium for 12 h and then incubated with 1mM H_2_O_2_ for 2 h. NPS, EPS and DPS: cultured with normal medium containing 5% (v/v) NPS, 5% EPS or 5% DPS respectively for 12 h and followed by 2 h incubation with 1mM H_2_O_2_. In the experiments with kinase inhibitors, U0126 (Cell Signaling Technology, Boston, MA, USA) or LY294002 (Cell Signaling Technology, Boston, MA, USA) was added 1 h before the addition of the sera.

### MTT method

Cells’ viability was determined by a MTT (4,5- dimethyl- 2- thiazolyl)- 2,5- diphenyl- 2- H- tetrazolium bromide, Sigma-Aldrich, St. Louis, MO) assay. The MTT powder was dissolved (5 mg/ml) into phosphate buffered saline (PBS) and sterilized through a 0.22-μm filter before use. HUVECs were seeded in a 96-well plate (5,000 cells/well, total 100 μl) and cultured overnight. At the end of the treatments, 10μl of the MTT solution was added to each well and incubated with 5% CO_2_ at 37°C for 4 h. Finally, the MTT solution was replaced by 150 μl dimethyl sulfoxide and the plate was incubated at room temperature for 15 min. The absorbance was measured at 570 nm by a microplate reader. Cells’ viability was expressed as the ratio of optical density (OD) with OD value of the control as 100%. All experiments were performed in six wells and repeated for three times.

### Measurements of malondialdehyde and activities of antioxidases

The medium level of malondialdehyde (MDA), as an indicator of lipid peroxidation, was assessed by the thiobarbituric acid reactive substances (TBARS) method using commercial kit (Nanjing Jiancheng Biotechnology Institute, China) according to the manufacturer’s instructions. The absorbance was measured at 532 nm using spectrophotometer.

The activities of total superoxide dismutase (SOD), catalase (CAT) and glutathione peroxidase (GSH-Px) in cell lysate were measured using kits (Nanjing Jiancheng Biotechnology Institute, China). Total SOD activity was spectrophotometrically quantified at 550 nm according manufacturer’s instruction. CAT activity was measured at 240 nm by analyzing the rate at which it caused the decomposition of H_2_O_2_ at 25°C. GSH-Px activity was determined based on its catalyzation by the oxidation of reduced glutathione in the presence of cumene hydroperoxide. The generation of nicotinamide adenine dinucleotide phosphate was measured spectrophotometrically at 340 nm.

### Measurement of intracellular reactive oxygen species

Intracellular reactive oxygen species (ROS) was evaluated by measuring changes in the fluorescence intensity in HUVECs preloaded with a ROS probe dihydroethidium (DHE, Invitrogen Life Technologies, Carlsbad, CA, USA). Three visual areas were randomly selected in each sample and the images of fluorescence were recorded by Nikon TES-2000s microscope. The software Image Pro Plus 5.0 was used to analyze the OD values. The data were expressed as the OD ratio with the OD values of the control as 100%.

### Immunofluorescence staining

HUVECs in logarithmic phase were seeded into 24-well chamber slides. After the treatments for the indicated times, cells were fixed with 4% (w/v) paraformaldehyde for 20 min, rehydrated in PBS for 15 min, and permeabilized in 0.1% (w/v) Triton X-100 at room temperature for 10 min. After rinse with PBS, the cells were blocked with 5% BSA in PBS for 30 min at room temperature. The cells were incubated with primary antibody (Abcam, Cambridge, UK) at 4°C overnight followed by FITC-conjugated secondary antibody for 1 h at room temperature. The images of Nrf2 with FITC staining were captured using a confocal microscope (OLYMPUS plus confocal system fluoview Ver 3.0).

### Real-time reverse transcription–polymerase chain reaction (RT-PCR)

Total RNA was extracted using TRIzol reagent according to the manufacturer's instructions (Invitrogen Life Technologies, Carlsbad, CA, USA) and treated with DNase I before cDNA synthesis to remove DNA contamination. First-strand cDNA was generated using SuperScript III first-strand synthesis system as recommended by the manufacturer and stored at -20°C until use. Subsequently, real-time PCR was performed to determine the mRNA expression of CAT, SOD-1, SOD-2 and GSH-Px-1 using the LightCycler 2.0 system. Glyceraldehyde-3-phosphate dehydrogenase (GAPDH) was used as the reference gene. Primer 3 software was used to design the primer sequences. The primer sequences are listed in Table 1. The final volume of the PCR reaction mixture was 25 μl, which consisted of 12.5 μl 2×SYBR premixture, 0.5 μl 10 μM forward primer and reverse primer, 1μl cDNA, and 10.5 μl sterilized deionized water. The PCR cycling conditions were as follows: 95°C for 10 min, followed by 40 cycles of 95°C for 10 sec and 60°C for 30 sec. Data were collected during each cycle at the 60°C extension step. The amplification efficiency was tested in standard curves using serial cDNA dilutions. Amplification specificity was checked using melting curves. For the comparison between the two groups, the software calculated the variation in the cycle threshold (Ct) in the treated group compared to the cycle threshold (Ct) of the control group, and this was expressed as a logarithmic base (2) value based on the formula 2^-ΔΔCt^, which was calculated as follows: 2^-ΔΔCt^ with ΔΔCt = (Ct_antioxidases_ −Ct_GAPDH_) treat groups-(Ct_antioxidases_ −Ct_GAPDH_) control group.

**Table 1:** List of primers for qPCR analysis.

Gene	Up primer	Down primer	Product size
GAPDH	tccctgagctgaacgggaag	ggaggagtgggtgtcgctgt	217bp
SOD-1	cgagcagaaggaaagtaatgga	cacaccatctttgtcagcagtc	223bp
SOD-2	cgtgactttggttcctttgac	atttgtaagtgtccccgttcc	116bp
CAT	gcctttggctactttgaggtc	gatgaagaaaatgggggtgtta	225bp
GSH-Px-1	agtcggtgtatgccttctcg	tcgttcatctgggtgtagtcc	142bp

### Statistical analysis

All values are expressed as means ± SEM. All date were first tested normality and homogeneity of variance. If these tests were passed, one-way ANOVA followed by Scheffe’s post-hoc test was performed to determine whether there were significant differences (*P* < 0.05) among groups and between two groups respectively. Otherwise, non-parametric analysis (Kruskal-Wallis test followed by mann-whitney *U* test) was performed. For all statistical comparisons, a *P* value < 0.05 was accepted to indicate significant differences.

## Results

### Rat LIPC sera protected H_2_O_2_–injured HUVECs

In pilot experiments searching for the optimal concentration of rat serum to be added to the culture medium, MTT assays were performed to evaluate the effects of different concentrations (0%, 5%, 10%, 20%) of rat serum on HUVECs proliferation with fetal calf serum as control. The maximum concentration (supplementation with 5%) of rat serum that did not induce cell proliferation was chosen.

**Fig 1:**
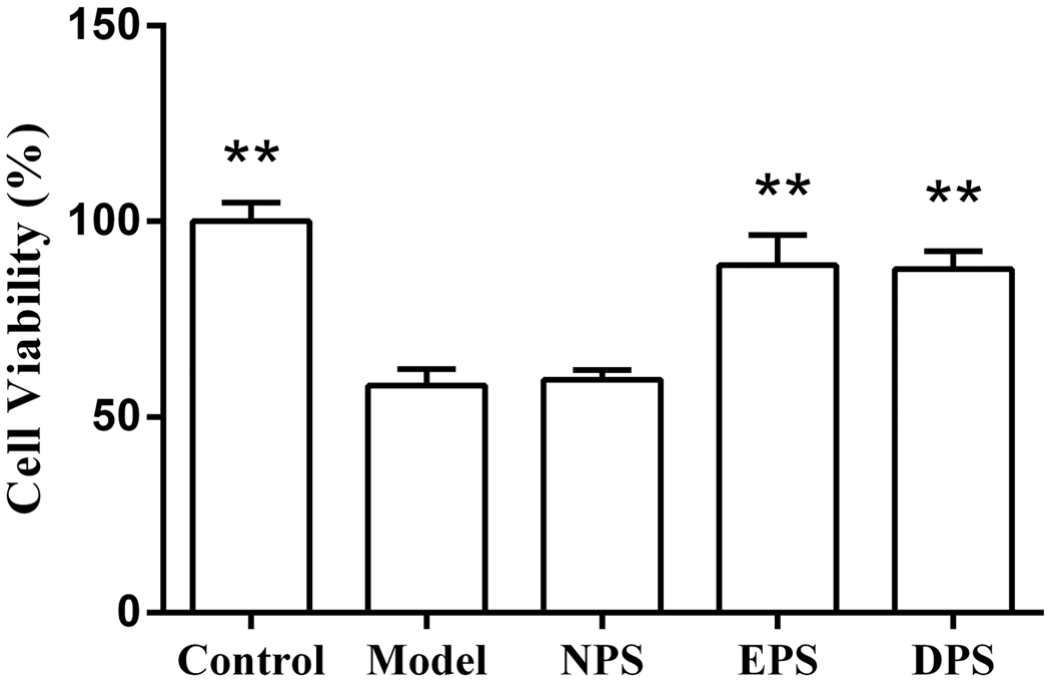
Rat LIPC sera increased the cells’ viability of H_2_O_2_-injured HUVECs. HUVECs were pretreated with 5% different sera for 12 h and followed by 2 h incubation with 1 mMH_**2**_O_**2**_. Cells’ viability was measured by MTT assay and expressed as the ratio of optical density (OD) with OD value of the control as 100%. The data (mean ± SEM) were obtained from at least three independent experiments, **P < 0.01 vs model. Control: cultured with normal medium without any intervention throughout the experiment. Model: cultured with normal medium for 12 h and then incubated with 1mM H_**2**_O_**2**_ for 2 h. NPS, EPS and DPS: cultured with normal medium containing either 5% NPS (serum derived from rats after sham LIPC), 5% EPS (serum derived from rats 20 min after LIPC) or 5% DPS (serum derived from rats 24 h after LIPC) respectively for 12 h and followed by 2 h incubation with 1mM H_**2**_O_**2**_. The figure legends are same in following figures unless illustrated elsewhere.

The cells incubated with 1mM H_2_O_2_ were found rounding, shriveling and detached from the growth surface. The cells’ viability in H_2_O_2_ model was significant reduced to 58.10 ± 4.27% (*P* < 0.01, Fig 1). The cells’ viabilities of EPS- and DPS-treated HUVECs were 88.76 ± 7.78% and 87.76 ± 4.65%, respectively, significantly higher than the model (*P* < 0.01). The cells’ viability of NPS-treated HUVECs was not significantly affected as compared with H_2_O_2_ model (59.56 ± 2.51% vs 58.10 ± 4.27%, *P* > 0.05).

### Rat LIPC sera reduced ROS in H_2_O_2_-injured HUVECs

**Fig 2:**
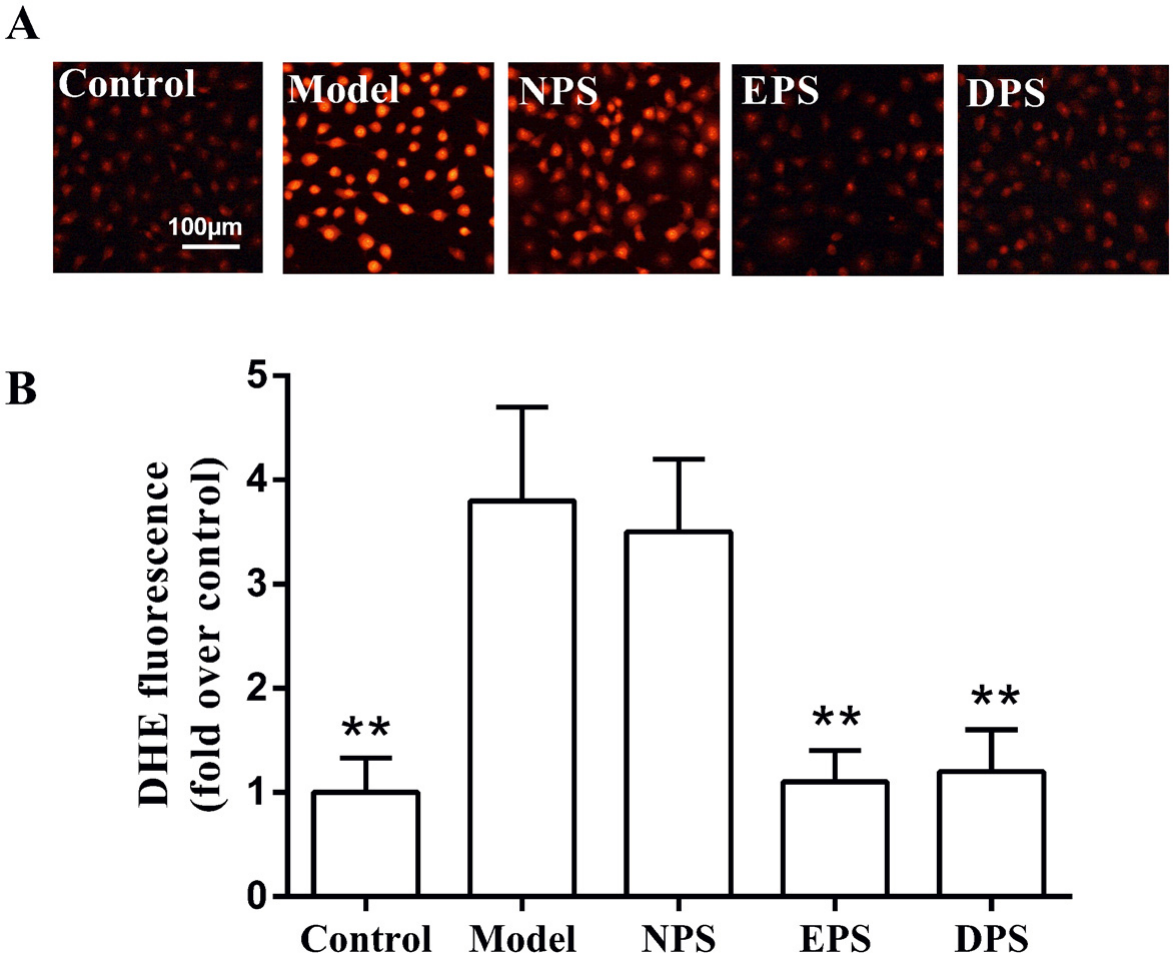
Rat LIPC sera decreased ROS in H_2_O_2_-injured HUVECs. ROS level was determined by measuring the intensity of dihydroethidium (DHE) fluorescence. The relative fluorescence intensities of DHE were analyzed by Image-Pro Plus software taking the fluorescence intensity of the control as 100%. A: original images of the cells preloaded with DHE. B: pooled data are expressed as mean ± SEM, n = 5, **P < 0.01 vs model.

Incubation with H_2_O_2_ for 2 h strikingly increased DHE fluorescence intensity (3.8 ± 0.9 fold of control, *P* < 0.01, Fig 2). Compared with H_2_O_2_ model, pretreatment with 5% EPS or 5% DPS for 12 h significantly reduced DHE fluorescence intensity to 1.1 ± 0.3 and 1.2 ± 0.4 fold of control (*P* < 0.01), respectively. However, NPS had no significant effect on the fluorescence intensification induced by H_2_O_2_ in HUVECs (3.5 ± 0.7 vs 3.8 ± 0.9 fold of control, *P* > 0.05).

### Rat LIPC sera decreased MDA and increased activities of antioxidases in H_2_O_2_-injured HUVECs

**Fig 3:**
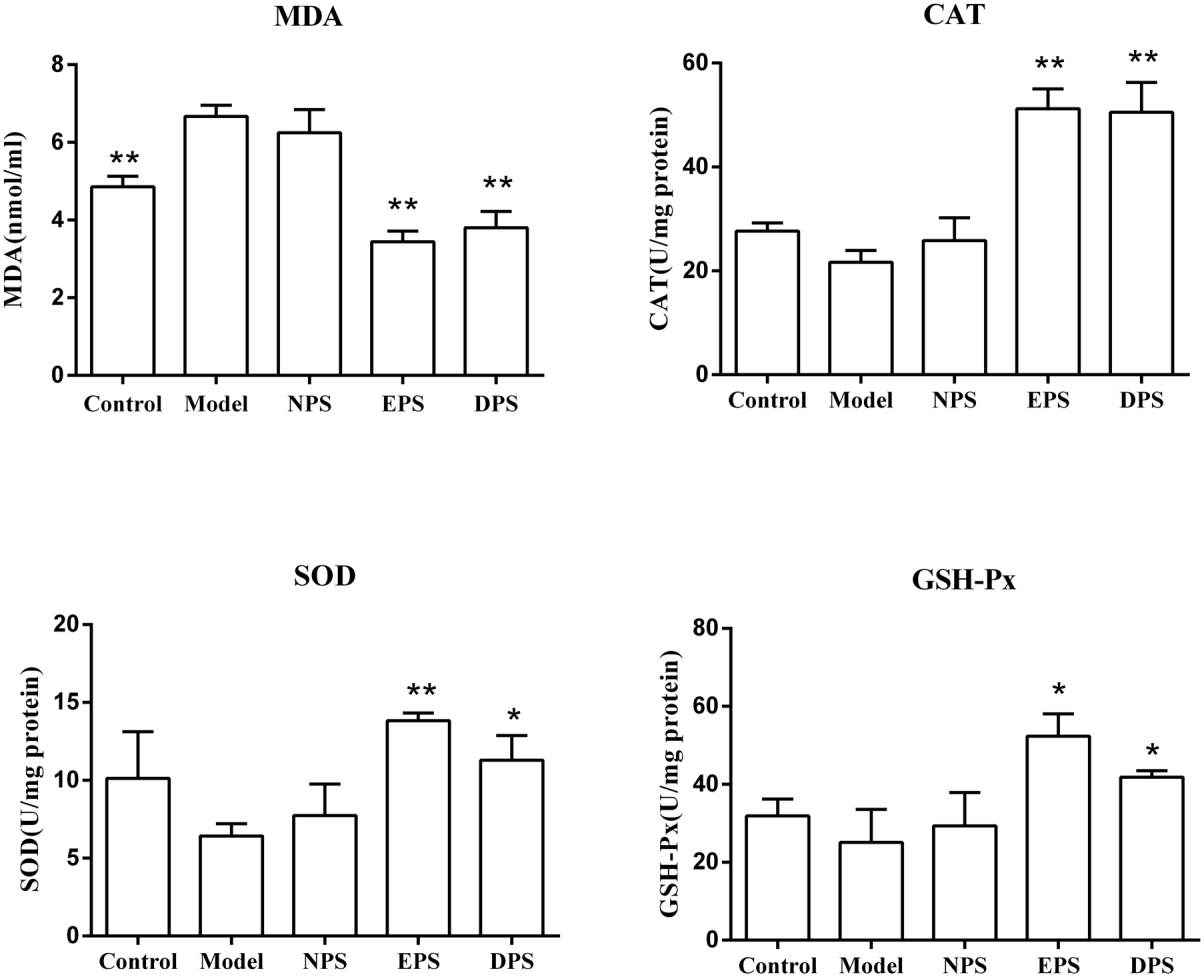
Rat LIPC sera reduced MDA and increased the activities of antioxidases in H_2_O_2_-injured HUVECs. Medium MDA concentration was measured spectrophotometrically at 532 nm. The activities of CAT, GSH-Px and total SOD were analyzed spectrophotometrically at 240 nm, 340 nm and 550 nm respectively. All data are expressed as mean ± SEM, n = 6, *P < 0.05, **P < 0.01 vs model.

The medium level of MDA is routinely used to reflect the extents of cell membrane damage attacked by H_2_O_2_ [16, 17]. Fig 3 shows that incubation with 1 mM H_2_O_2_ for 2 h increased the medium MDA concentration from 4.86 ± 0.28 nmol/ml to 6.67 ± 0.29 nmol/ml (*P* < 0.01). Compared with H_2_O_2_ model, MDA levels in EPS and DPS were reduced to 3.44 ± 0.27 nmol/ml (*P* < 0.01) and 3.80 ± 0.41 nmol/ml (*P* < 0.01). NPS had no significant effect on MDA level in H_2_O_2_-injured HUVECs (6.25 ± 0.59 nmol/ml vs 6.67 ± 0.29 nmol/ml, *P* > 0.05). Enzymatic study showed that H_2_O_2_ incubation did not significantly affected the endothelial intracellular activities of CAT, GSH-Px and SOD, while preincubation with EPS or DPS, but not NPS, significantly elevated the activities of the antioxidases (*P* < 0.01 vs model).

### Rat LIPC sera upregulated the expression of antioxidases

**Fig 4:**
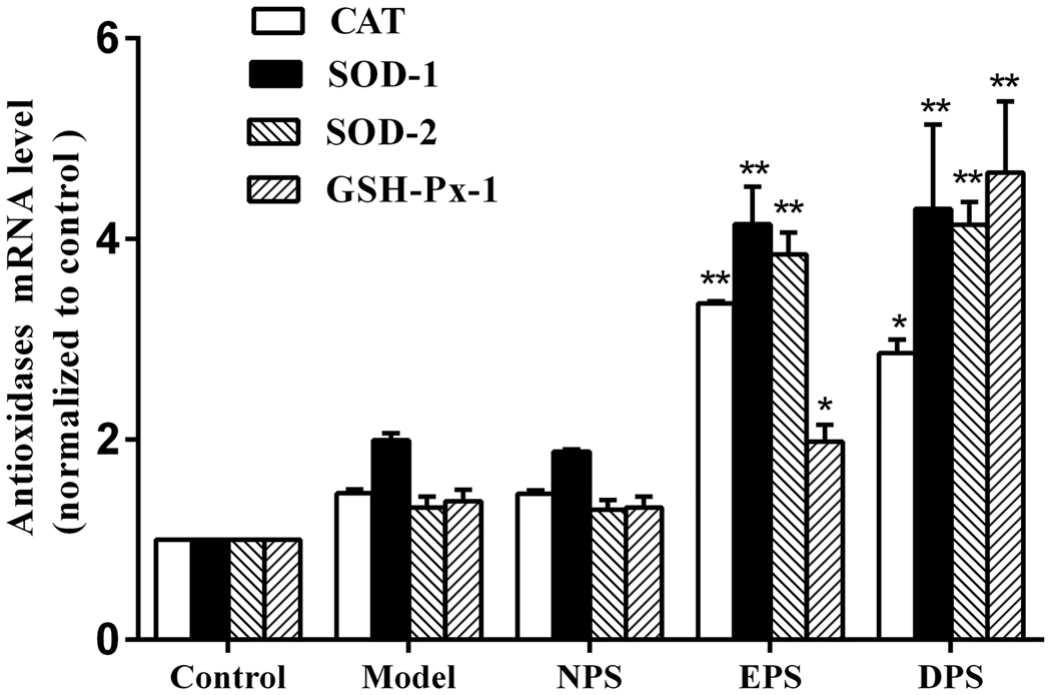
Rat LIPC sera upregulated the mRNA expression of antioxidases in HUVECs. CAT, SOD-1, SOD-2 and GSH-Px-1 mRNA levels in HUVECs were detected by real-time PCR. The mRAN expression of antioxidases was presented by normalizing the antioxidases expression with GAPDH and taking control as 100%. All data are expressed as mean ± SEM, n = 6, *P < 0.05, **P < 0.01 vs model.

Real-time PCR study showed that incubation with either 5% EPS or 5% DPS, but not 5% NPS, significantly increased the mRNA levels of the antioxidases (CAT, SOD-1, SOD-2, GSH-Px-1), compared with model (Fig 4). Statistical analysis revealed that EPS and DPS were almost equipotent on the expression of CAT, SOD-1, SOD-2, but EPS on GSH-Px-1 was weaker than DPS (1.97 ± 0.17 vs 4.66 ± 0.71, *P* < 0.01).

### Rat LIPC sera enhanced nuclear factor E2-related factor 2 (Nrf2) translocation in H_2_O_2_-injured HUVECs

**Fig 5:**
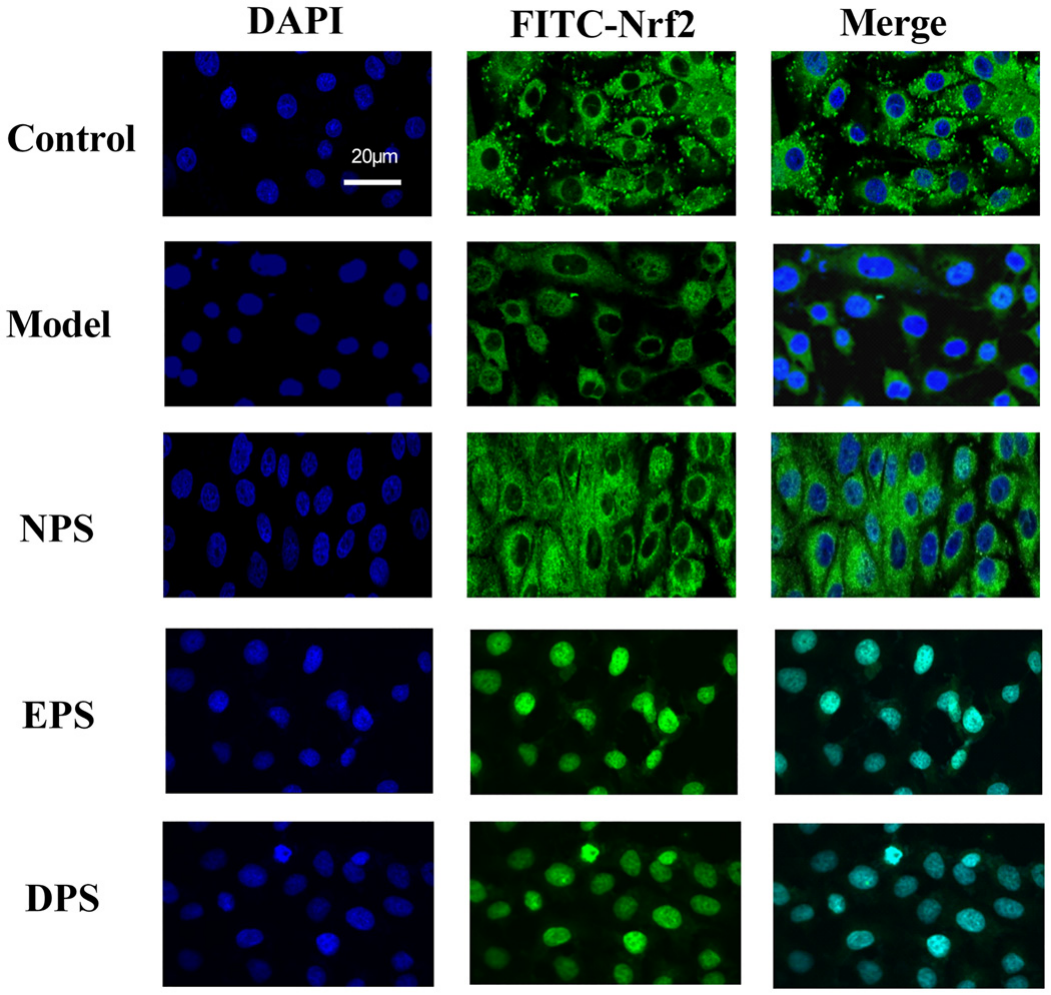
Rat LIPC sera enhanced Nrf2 localization into nucleus inH_2_O_2_-injured HUVECs. Localization of Nrf2 was performed by immunofluorescence and confocal microscopy. Nrf2 was stained with an anti-Nrf2 antibody and visualized with a secondary antibody conjugated with FITC (green). The nuclei were counterstained with DAPI (4’,-diamidino-2-phenylindole) staining indicating the location of the nucleus (blue). The merged image showed the nuclear location of Nrf2 protein.

Nrf2 is a transcription factor that regulates the expression of cytoprotective genes in response to oxidative stress [18]. As the translocation of Nrf2 into the nucleus is critical for its activation, the distribution of Nrf2 in HUVECs was examined by immunofluorescence in the present study. Fig 5 shows that the pattern of Nrf2 distribution between nucleus and cytoplasm was not significantly different in control, model and NPS. Pretreatment with EPS or DPS significantly intensified the positive signals of Nrf2 in the nucleus.

### Effects of rat LIPC sera on Nrf2 translocation and cells’ viability were affected by neither PI3K/Akt inhibitor nor MEK/ERK inhibitor in H_2_O_2_-injured HUVECs

**Fig 6:**
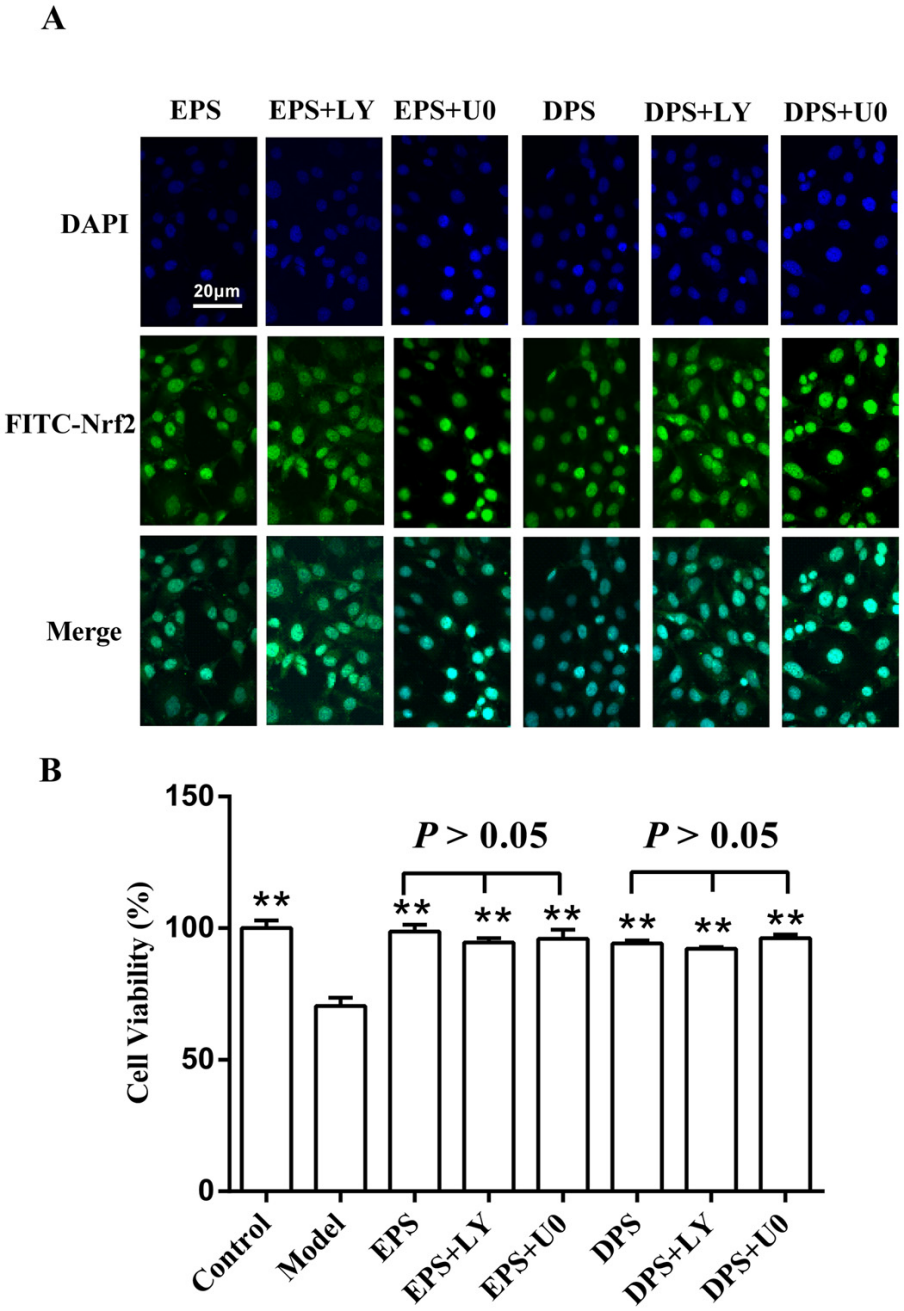
Effects of rat LIPC sera on Nrf2 translocation and cellular viability were affected by neither PI3K/Akt inhibitor nor MEK/ERK inhibitor in H_2_O_2_-injured HUVECs. The cells were cultured in medium for 24 h, after which the inhibitors U0126 (U0, 26 μM) and LY294002 (LY, 25 μM) were added 1 h before the especial serum and H_**2**_O_**2**_ treatments. A: nucleus localization of Nrf2 determined by immunofluorescence and confocal microscopy. B: cells’ viability detected using MTT method. The data (mean ± SEM) were obtained from at least three independent experiments, **P < 0.01 vs model.

Since it has been reported that ischemic preconditioning activates the MEK/ERK and PI3K/AKT signaling pathway [6, 19], we studied whether PI3K/Akt and MEK/ERK signaling pathways were involved in LIPC-induced protection. The cells were pretreated with a MEK/ERK inhibitor U0126 (26 μM) or a PI3K/Akt inhibitor LY294002 (25 μM) for 1 h prior to the addition of the sera. The results showed that neither LY294002 nor U0126 significantly affected the effects of rat LIPC sera on Nrf2 translocation (Fig 6A) and cells’ viability in H_2_O_2_-injured HUVECs (Fig 6B).

## Discussion

The major findings of the present study are (1) LIPC protects the HUVECs from the oxidative stress injury induced by H_2_O_2_; (2) the Nrf2 translocation and upregulation of antioxidases including CAT, SOD-1, SOD-2 and GSH-Px-1 are involved in the protection.

Vascular endothelial cells widely distribute in the body and play an irreplaceable role in vascular homeostasis. Endothelium dysfunction is, in most cases, preluded with production of excessive amounts of ROS [20, 21], which appears inevitably in hypertension [22], hyperlipidemia, obesity [23], diabetes [24], ischemia, atherosclerosis and other vascular disorders [25]. Protection of endothelium from oxidative stress with antioxidant drugs is suggested as a promising treatment for cardiovascular diseases [20]. Antioxidative effects were suggested responsible, at least partially, for LIPC-induced protection on injured parenchymal cells of the heart [5, 11] and liver [6]. It has also demonstrated that LIPC is beneficial to endothelium-dependent vasodilation in a number of conditions [1–3, 13, 15]. However, how LIPC affects redox state of endothelium remains largely unknown. The present experiments mimicked oxidative stress by applying H_2_O_2_ in cultured HUVECs and studied the protective effects of LIPC. The present study demonstrated that the rat EPS and DPS protected the HUVECs from oxidant stress injury. These results were in consistence with reported results that the cardioprotection of LIPC was transferable across species [12] and that LIPC prevented endothelial I/R injury in conduit vessels with two temporally distinct phases of protection in human in vivo [2].

Based on the fact that inhibition of excessive production of ROS and reinforcement of cellular antioxidant capability protect the cells from the oxidative stress injury, we supposed that LIPC may protect HUVECs by upregulating antioxidases. Nrf2 is a key transcription factor that regulates intracellular redox balance and inflammation through activation of its targeting genes encoding antioxidant and detoxifying molecules [26, 27]. Under normal conditions, Nrf2 binds to Kelch-like erythroid cell-derived protein 1 (Keap1), a cytosolic repressor protein, that disenables Nrf2 translocation from cytoplasm to the nucleus. Upon activation, the Keap1/Nrf2 complex is disaggregated and Nrf2 is released from Keap1 and translocates to the nucleus, where it binds to antioxidant response element (ARE) to induce expression of antioxidant enzymes, which include heme oxygenase-1, SOD, GSH-Px, CAT, NAD(P)H: quinine oxidoreductase 1, thioredoxin-1, etc [28, 29]. Many studies have showed that the activation of Nrf2 was involved in the protection of vascular endothelium [30–32]. The present study demonstrated that pretreatment of HUVECs with EPS or DPS enhanced the translocation of Nrf2 from cytoplasm to nucleus, and markedly increased mRNA levels of antioxidases including CAT, SOD-1, SOD-2, GSH-Px-1. These results suggested that LIPC protected HUVECs from H_2_O_2_-induced injury via the Nrf2-ARE axis.

Although the detailed mechanisms via which LIPC confers the cellular protection remains unclarified, it has been suggested that LIPC activated the MEK/ERK in liver cells [6] and PI3K/AKT in cardiac cells [33]. Interestingly, a recent study showed that human LIPC plasma protects HUVECs from hypoxia-induced cell damage with increased phosphorylation of ERK-1/2 [34]. However, in the present study, neither MEK/ERK inhibitor U0126 nor PI3K/AKT inhibitor LY294002 significantly affected the LIPC-induced protective effects and enhancement of Nrf2 translocation. The differences among the reported and our results may be presumably due to following factors: (1) used kinase inhibitors have limited specificity towards the oriented target kinase [35]; (2) observed biologic phenomena may, in almost every case, be results of intricate net regulation rather than of just one signaling pathway; (3) LIPC may be pleiotropic. Further studies are needed to explore the molecular mechanisms underlying LIPC-induced cytoprotection.

In conclusion, the present study demonstrates that LIPC protects HUVECs from H_2_O_2_-induced damage and suggests that Nrf2 translocation and the upregulation of its downstream antioxidase expression may be involved in the cytoprotection.
